# Analysis of the improvement in monocular amblyopia visual acuity caused by the changes in non-amblyopia visual acuity in 74 adults

**DOI:** 10.1097/MD.0000000000034606

**Published:** 2023-09-15

**Authors:** Yong Guo, Hong Yan, Chenjun Guo, Dan Zhang, Jue Wang, Yan Li, Yuhuan Yang

**Affiliations:** a Xi'an Bright Eye hospital, Xi’an, China; b Xi’an People’s Hospital (Xi’an Fourth Hospital), Shaanxi Eye Hospital, Affiliated Grangren Hospital of School of Medicine, Xi’an Jiaotong University, Xi’an, China; c Tangdu Hospital of Air Force Military Medical University, Xi’an, China.

**Keywords:** adult amblyopia, monocular amblyopia, nerve plasticity, pattern visual evoked potential, visual central

## Abstract

To observe the clinical phenomenon of amblyopia vision improvement in patients with monocular amblyopia over 18 years old after non-amblyopia diseases, analyze the conditions and causes of vision improvement, explore the plasticity of the adult optic nerve, and provide a clinical basis for the treatment of adult amblyopia. A total of 74 patients with monocular amblyopia combined with non-amblyopia visual acuity decline from 2018 to 2021 were collected. The patient’s age, initial best-corrected visual acuity (BCVA), pattern visual evoked potential examination results, and visual acuity regression were recorded. The BCVA of amblyopia was recorded every 3 months using an early treatment of diabetic retinopathy study visual acuity chart. In the 3rd month, BCVA increased by 16.2%, reaching 98% in the 9th month and 100% in the 12th months. According to the age of patients, the group aged 18 to 35 years was better than the group aged 35 to 60 years, whereas the group aged 35 to 60 years was better than the group aged over 60 years (*P* < .05). According to the comparison of initial visual acuity, the BCVA of the < 5 letter group was lower than that of the other 2 groups (*P* < .05). According to the pattern visual evoked potential results, the peak time of the < 10 ms group was better than that of the 10 to 20 ms group; the 10 to 20 ms group was better than that of the > 20 ms group; the peak decrease of the < 30% group was better than that of the 30% to 50% group; and the 30% to 50% group was better than that of the > 50% group (*P* < .05). The visual acuity regression of amblyopia in the 0.5 to 1-year group was higher than that in the other 2 groups (*P* < .05). This study confirms that adult amblyopia can still be cured under certain conditions. This visual plasticity is related to age, initial visual acuity, and excitability of the visual center. This study provides new clinical evidence and diagnostic ideas for the study of the pathogenesis of adult amblyopia.

## 1. Introduction

Ophthalmologists agree that patients with amblyopia can be effectively treated during childhood.^[1–3]^ Once the children are over a certain age, amblyopia treatment becomes ineffective. Some ophthalmologists believe that beyond the age of 6 or 7 years, amblyopia is less likely to respond to treatment, whereas other ophthalmologists believe that this age can be delayed until 9 or 10 years.^[4–6]^ The age limit for the successful treatment of amblyopia recommended by the American

Academy of Ophthalmology is <10 years.^[7]^ The idea of an age limit for amblyopia treatment comes from people’s cognition of visual development, and the “critical period” of human visual development ends at the age of 6 years to 7 years.^[8]^ However, this view has not been confirmed by sufficient clinical data. Numerous case reports have demonstrated that adults over 18 years old respond to amblyopia treatment.^[9,10]^ This study retrospectively observed adult cases of monocular amblyopia. When the disease developed in the non-amblyopic eye, interocular visual suppression was terminated. The visual changes and characteristics of the amblyopic eye were recorded, and the effective age range and sensitive factors for amblyopia treatment were analyzed. The mechanism of visual development provides new clinical evidence and diagnostic ideas for the diagnosis and treatment of amblyopia.

## 2. Materials and methods

### 2.1. Research objects

This study retrospectively analyzed 74 patients (See Table S1, Supplemental Digital Content, http://links.lww.com/MD/J468, which illustrates all the initial and transitioned data on amblyopia eyes, including the visual acuity, and causes of amblyopia) diagnosed with monocular amblyopia at the Tangdu Hospital of Air Force Military Medical University and Xi’an Purui Eye Hospital from June 2018 to June 2021. All of them signed informed consent forms. This study passed the ethical review of the Ethics Committee of Xi’an Purui Eye Hospital. In addition, patients who had any eye disease in the other eye with lower visual acuity than the amblyopic eye were also analyzed. Among them, males and females accounted for 54.1% (40/74) and 45.9% (34/74), respectively. All patients were over 18 years old, 9 were 18 to 30 years old, 12 were 30 to 40 years old, and 15 were 40 to 50 years old. A total of 17 patients were 50 to 60 years old, and 21 patients were > 60 years old. The patients were divided into 3 groups according to age: Group A (18–35), Group B (35–60), and Group C (over 60 years). According to the patients’ initial visual function status, all patients were divided into Group D (best-corrected visual acuity [BCVA] of < 5), Group E (5–10), and Group F (> 20 letter). According to the pattern visual evoked potential (PVEP) examination results of the amblyopic eyes, all patients were divided into Group G (PVEP peak delay < 10), Group H (10–20), and Group I (> 20 ms). According to the results of the PVEP examination, all patients were divided into Group J (with a peak PVEP decrease of < 30%), Group K (30%–50%), and Group L (> 50%). All patients were divided into Group M (0.5–1 years), Group N (1–1.5 years), and Group O (1.5–2 years) according to the time of visual impairment in non-amblyopic eyes. Furthermore, 49 patients were diagnosed with cataracts in the other eye, 5 patients were diagnosed with retinal detachment, 7 patients were diagnosed with vitreous hemorrhage, 2 patients were diagnosed with a retinal contusion, and 11 patients were diagnosed with macular degeneration (Table 1). All patients underwent slit-lamp examinations in both eyes to exclude keratopathy and previous surgery. Fundus, intraocular pressure, and visual acuity examinations were performed on all patients.

**Table 1:** Structural composition of amblyopia patients (*n*).

Patient age (yr)	Total	18–30	30–40	40–50	50–60	>60
Number of people	74	9	12	15	17	21
Proportion (100%)		12.2	16.2	20.3	23.0	28.4
Non-amblyopic eye disease		Cataract	Retinal detachment	Vitreous hemorrhage	Eye trauma	Macular degeneration
Number of people	74	49	5	7	2	11
Proportion (100%)		66.2	6.8	9.5	2.7	14.8

### 2.2. Inspection method

The conjunctiva, cornea, anterior chamber, pupil, and lens of all eyes were examined by the deputy chief physician of the ophthalmology general clinic using a German Zeiss SL30 slit microscope. The patient’s pupils were dilated by applying midorie eye liquid 3 times for 5 minutes each. The pupils were dilated to 8 mm in diameter, and both eyes were photographed with Zeiss Clarus 500 fundus photography. All eyes were examined by PVEP using the GT-2008V visual electrophysiology instrument of the Chongqing Guote Medical Company. The stimulus source was selected to flip the checkerboard, and the signals were superimposed 64 times. The examination was performed by the same senior special examiner and completed by the professional attending physician. The reading and diagnosis of fundus photography and visual electrophysiological examination results were completed by the same professional deputy chief physician of the fundus. Amblyopic eyes underwent medical optometry once a month, and optometry was simultaneously completed by 2 senior optometrists. The visual acuity examination employed the early treatment of diabetic retinopathy study vision chart to record the BCVA of the amblyopic eye. Stereoscopic function examination was performed using the Titmus album (fly only), and the eye position examination was measured using the prism + covering method. The reading of optometry results and the diagnosis of amblyopic eyes were completed by the same deputy chief physician of optometry. Before comparing the groups, we conducted a balance test to ensure that there were no significant differences in other factors. We found that there was no statistical difference among the groups.

### 2.3. Observation indicators

When the visual acuity of the patient’s non-amblyopic eye was lower than that of the amblyopic eye, the changes in the patient’s binocular vision were recorded, and medical optometry and early treatment of diabetic retinopathy study visual inspection were performed every month. When BCVA improved by ≧ 2 letters, it was recorded as improved vision. When the BCVA decreased by ≧ 2 letters, it was recorded as visual acuity regression, and when the non-amblyopic eye disease was cured, the observation was excluded. The recorded metrics were as follows: Dynamic changes in visual function in adults with amblyopia; A comparison of the relationship between the changes in amblyopic visual function and the patient’s age, initial visual acuity, PVEP examination, and visual impairment time of non-amblyopic eyes; and The visual function of the regressed amblyopic eyes after the non-amblyopic eyes were treated.

### 2.4. Statistical methods

Statistical analysis was performed using the SPSS27 software. The data were initially tested for normality. The data that met the normal distribution were subjected to 1-way ANOVA, and the data that did not satisfy the conditions were tested using nonparametric tests. All statistics used *P* < .05 as the significance test standard.

## 3. Results

### 3.1. Relationship between visual acuity improvement and age in amblyopic eyes

**Figure 1.**
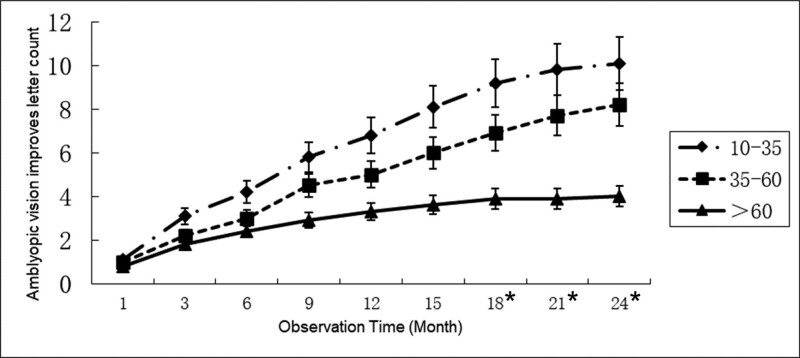
Relationship between improvement in the visual acuity of amblyopia and age. The 18–35 year-old group is better than the 35–60 year-old group. The 35–60 year-old group is better than the older than 60-year-old group. * Indicates that the difference between the 3 groups is statistically significant (*P* < .05).

The visual function changes of the amblyopic eyes of the Group A, Group B, and Group C were compared, and the observation was terminated when the non-amblyopic eye disease was cured. The optometry results of the patients showed that the improvement in the BCVA of patients with amblyopia Group A was significantly better than that of the other 2 groups. The Group B was better than the older than Group C, and after 18 months of observation, the difference between the 3 groups was statistically significant (*P* < .05) (Fig. 1).

### 3.2. Relationship between visual acuity improvement and initial visual acuity in amblyopic eyes

**Figure 2.**
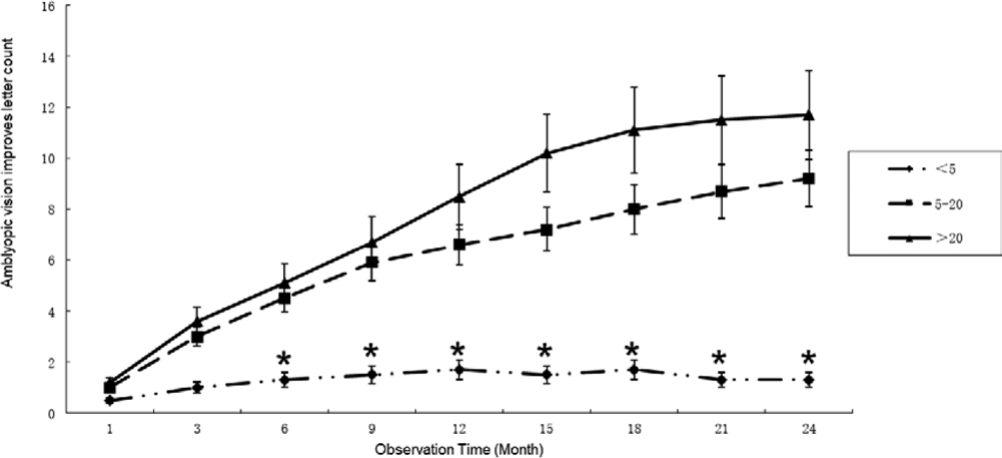
Relationship between improvement in the visual acuity of amblyopia and initial visual acuity. The < 5 letter group is lower than the other two groups. * Indicates that the difference between the < 5 letter group and the other two groups is statistically significant (*P* < .05).

The visual function changes of the amblyopic eyes of Group D, Group E, and Group F were compared, and the observation was terminated when the non-amblyopic eye disease was cured. The optometry results of the patients showed that the improvement of the BCVA of the amblyopic eyes in the Group D was significantly lower than that of the other 2 groups, and the Group F was better than the Group E. After 6 months of observation, the Group D was better than the other 2 groups. A significant difference was found between the 2 groups, and the difference was statistically significant (*P* < .05). The difference between the Group E and the Group F was not statistically significant (*P* > .05) (Fig. 2).

### 3.3. Relationship between visual acuity improvement and PVEP in amblyopic eyes

**Figure 3.**
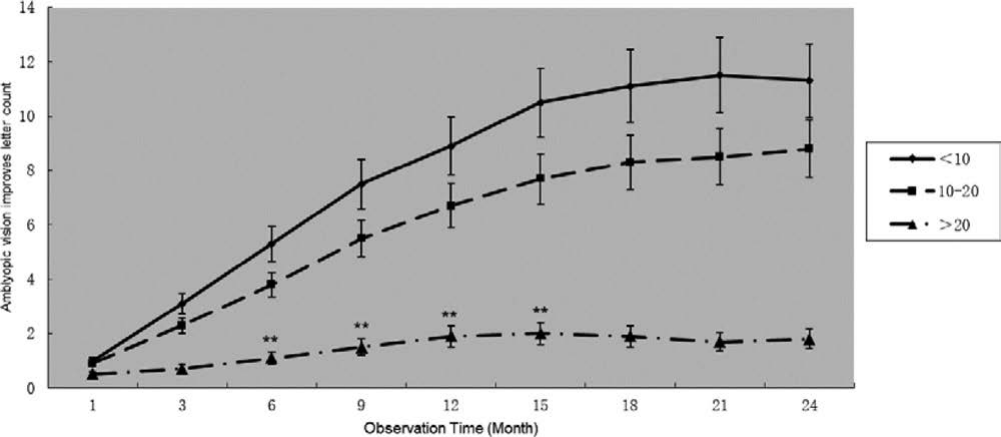
The relationship between improvement of the visual acuity of amblyopia and peak time of pattern visual evoked potential. The < 10 ms group is better than the 10–20 ms group, and the 10–20 ms group is better than the > 20 ms group. ** Indicates that the difference between the < 5 letter group and the other two groups is statistically significant (*P* < .05), and * indicates that the difference between the 3 groups is statistically significant (*P* < .05).

The PVEP results of the non-amblyopic eyes were related to primary eye disease; however, no correlation was observed between the binocular data. The visual function changes of the amblyopic eyes of Group G, Group H, and Group I were compared, and the observation was terminated when the non-amblyopic eye disease was cured. The optometry results of the patients showed that the improvement of the BCVA of patients with amblyopia in the Group I was significantly worse than that of the other 2 groups, and the improvement of the BCVA of the patients in the Group G was better than that of the Group H. The difference was statistically significant (*P* < .05). After 18 months of observation, a significant difference was found between the 3 groups, and the difference was statistically significant (*P* < .05) (Fig. 3).

**Figure 4.**
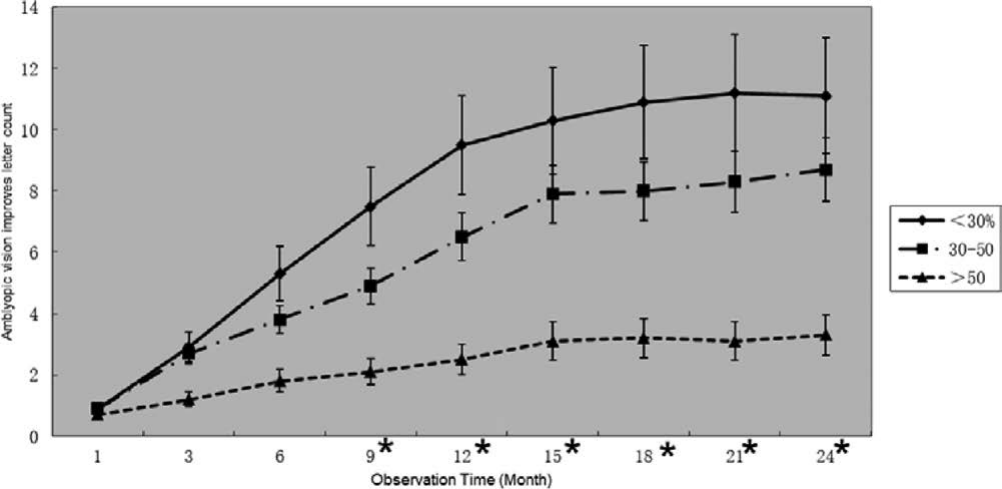
The relationship between improvement of the visual acuity of amblyopia and peak value of pattern visual evoked potential. The < 30% group is better than the 30%–50% group, and the 30%–50% group is better than the > 50% group. * Indicates that the difference between the 3 groups is statistically significant (*P* < .05).

The visual function changes of the amblyopic eyes of Group J, Group, K and Group L were compared, and the observation was terminated when the non-amblyopic eye disease was cured. The optometry results of the patients showed that the Group L in the amblyopic group, with significantly worse improvement in the BCVA than the other 2 groups, and the Group J was better than the Group K. After 9 months of observation, a significant difference was found between the 3 groups. The difference was statistically significant (*P* < .05) (Fig. 4).

### 3.4. Relationship between visual acuity improvement in amblyopic eyes and time of visual impairment in the fellow eye.

**Figure 5.**
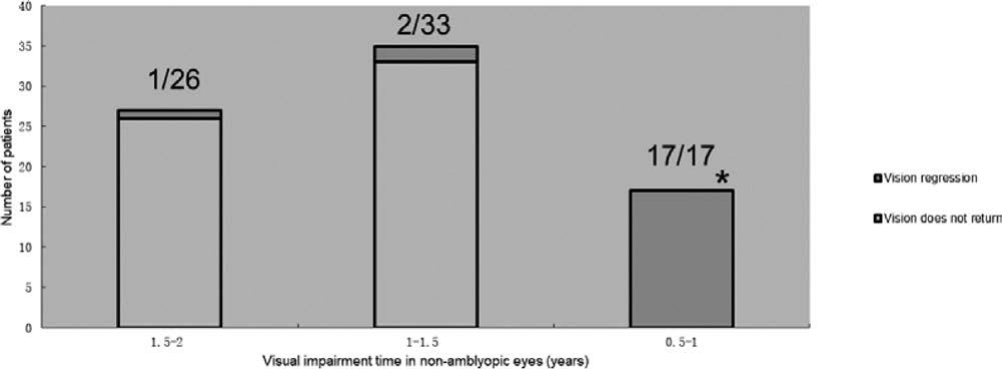
Relationship between visual acuity regression of amblyopia and visual impairment time of non-amblyopia. The 0.5–1-year group is higher than the 1–1.5-year group, and the 1–1.5-year group is higher than the 1.5–2-year group. * Indicates that a statistically significant difference is observed between the other two groups (*P* < .05).

When the non-amblyopic eye disease was cured, the recording of the visual acuity of the amblyopic eye was continued. From the 6th month, visual acuity regression occurred, and 27% (20/74) of the patients experienced visual acuity regression (Table 2). The visual function regression of the amblyopic eyes in Group M, Group N and Group O was compared. The optometry results of the patients showed that the visual acuity regression of the amblyopic eyes in the Group M was significantly higher than that in the other 2 groups, and the Group N was higher than the Group O. Among them, the Group M was significantly different from the other 2 groups, and the difference was statistically significant (*P* < .05). The difference between the Group O and the Group N was not statistically significant (*P* > .05) (Fig. 5).

**Table 2:** Improvement of the visual acuity of amblyopia (*n*).

Observation time (mo)	1	3	6	9	12	15	18	21	24
Improved vision	1	12	47	51	48	42	40	38	31
Number of observers	74	74	61	52	48	42	40	38	31
Proportion (100%)	1.4	16.2	77	98	100	100	100	100	100

## 4. Discussion

Amblyopia is a disorder of the visual cortex in which patients present with a unilateral or bilateral decrease in BCVA that cannot be explained by other ocular disorders.^[11]^ Amblyopia in most patients is due to the inhibition of the visual signal to the amblyopic eye during visual development, which leads to the reduction of the signal reaching the visual center in the visual path of the amblyopic eye. Long-term visual signal reduction results in changes in the anatomy of the lateral geniculate nucleus and occipital cortex.^[12]^ Evidence suggests that the area and axons of the extra striatal regions in the optic radiation of the brain corresponding to the amblyopic eye are abnormal.^[13]^ Conventional wisdom holds that these abnormalities only occur during sensitive or critical periods of damage to the visual system when the visual center has sufficient plasticity, and cortical modifications are more likely to occur. Clinically, this period generally refers to birth to 9 years of age, but the exact age has not been proven.^[14]^ However, numerous animal and human case reports deny this view.^[15–17]^ Restoring the plasticity of the adult visual cortex and reducing the suppression of visual signals between the eyes have become research hotspots and have gained increasing attention.^[18,19]^ In this subject, the non-amblyopic eyes of the study population suffered from diseases and decreased visual acuity. The suppression of the visual signals of the amblyopic eyes by the healthy eyes was terminated, and the BCVA of the amblyopic eyes was significantly improved. The number of improvements reached 16.2% in the 3rd months, 98% in the 9th months, and 100% after the 12th months. The effective rate reaches or exceeds that of children with amblyopia under the age of 18 years. However, this finding does not suggest that adult optic neuroplasticity is superior to that of children. Patients with amblyopia who were selected for this study all had monocular amblyopia, and their other eyes developed a severe eye disease and decreased or even lost visual function. When the visual signal inhibition of the amblyopic eye by the non-amblyopic eye is completely terminated or even reversed, and the visual signal of the amblyopic eye becomes the main signal of the cerebral visual cortex, the therapeutic effect of amblyopia is significantly amplified, which is similar to our childhood. Coverage treatments are fundamentally different.

In this study, we grouped all the observed subjects by age and obtained similar results. The 18 to 35 year-old group was better than the 35 to 60-year-old group, and the 35 to 60-year-old group was better than the older than a 60-year-old group. The younger are patients with monocular amblyopia, and when the suppression of the visual signal of the amblyopic eye by the healthy eye disappears, the more obvious is the visual function of the amblyopic eye. In particular, patients over 60-year old were significantly worse than the other 2 groups. Evidently, this result may also be related to the decline in dopamine levels in the elderly.^[20,21]^ To explore the relationship between age and the incidence of amblyopia, we can give Levodopa (L-dopa) to patients over 60-year old and observe whether this age difference is reduced.

By recording the intensity range of transcranial magnetic stimulation, Tuna et al^[22]^ found that binocular visual deprivation can increase the excitability of the visual cortex for a short time, whereas monocular visual deprivation reduces the excitability of the visual cortex. However, Liu et al^[23]^ observed a change in VEP and found a contrasting result: short-term monocular visual deprivation can increase the VEP peak, whereas binocular visual deprivation decreases the VEP peak. In this study, we divided the observed subjects into 3 groups according to the PVEP peak and peak time. Patients with delayed PVEP peak and decreased peak had poor treatment effects for amblyopia. We considered this finding to be a long-term monocular visual deprivation, which reduces the plasticity of the visual cortex.

Interestingly, in this study, we found that the visual improvement in this amblyopic eye was not permanent. After the non-amblyopic eye was treated, some patients developed visual regression, which can reach 100% (17/17) within 1 year (Table 3). We consider these findings to be consistent with the features of the visual cortex. Pons et al^[24]^ proved that the excitation and inhibition of the visual cortex are reversible, and this reversibility is related to time. When the inhibition exceeds a certain limit, the plasticity of the visual cortex is turned off. This inhibition limit is impossible for adults with amblyopia. The key to effective healing and how to find and reverse this boundary have become the key to the treatment of amblyopia. This article has a small sample size, and all participants are of East Asian descent, so the results obtained cannot represent all ethnic groups. Due to the relatively long observation period, tracking became difficult, and there were cases of individual patients being lost to follow-up. The lack of examination methods for the intracranial segment of the visual pathway and the visual center hinders the exploration of the pathogenesis of amblyopia in the central visual system.

**Table 3:** Visual acuity declines of amblyopia (*n*).

Observation time (mo)	6	9	12	15	18	21	24
Vision regression	8	14	17	18	19	20	20
Vision does not return	66	60	57	56	55	54	54
The percentage of people falling back (100%)	10.8	18.9	23.0	24.3	34.5	27.0	27.0

## Author contributions

**Conceptualization:** Yong Guo, Yuhuan Yang.

**Data curation:** Yong Guo.

**Formal analysis:** Jue Wang.

**Funding acquisition:** Hong Yan, Jue Wang.

**Investigation:** Hong Yan, Chenjun Guo, Jue Wang.

**Methodology:** Chenjun Guo, Dan Zhang, Jue Wang.

**Project administration:** Chenjun Guo, Dan Zhang, Jue Wang, Yuhuan Yang.

**Resources:** Dan Zhang.

**Software:** Yuhuan Yang.

**Supervision:** Yuhuan Yang.

**Validation:** Yan Li, Yuhuan Yang.

**Visualization:** Yan Li.

**Writing – original draft:** Yan Li.

## Supplementary Material



## References

[1] Coco-Martin MB Piñero DP Leal-Vega L. The potential of virtual reality for inducing neuroplasticity in children with amblyopia. J Ophthalmol. 2020;2020:7067846. 32676202 10.1155/2020/7067846 PMC7341422

[2] Hernández-Rodríguez CJ Piñero DP. Active vision therapy for anisometropic amblyopia in children: a systematic review. J Ophthalmol. 2020;2020:4282316. 32733699 10.1155/2020/4282316 PMC7376429

[3] El Mallah MK Chakravarthy U Hart PM. Amblyopia: is visual loss permanent? Br J Ophthalmol. 2000;84:952–6. 10966943 10.1136/bjo.84.9.952 PMC1723664

[4] Park SH. Current management of childhood amblyopia. Korean J Ophthalmol. 2019;33:557–68. 31833253 10.3341/kjo.2019.0061 PMC6911788

[5] Gopal SKS Kelkar J Kelkar A. Simplified updates on the pathophysiology and recent developments in the treatment of amblyopia: a review. Indian J Ophthalmol. 2019;67:1392. 31436180 10.4103/ijo.IJO_11_19 PMC6727694

[6] Kaur S Bhatia I Beke N. Efficacy of part-time occlusion in amblyopia in Indian children. Indian J Ophthalmol. 2021;69:112–5. 33323591 10.4103/ijo.IJO_1439_19 PMC7926133

[7] Pineles SL Aakalu VK Hutchinson AK. Binocular treatment of amblyopia: a report by the American academy of ophthalmology. Ophthalmology. 2020;127:261–72. 31619356 10.1016/j.ophtha.2019.08.024

[8] Hensch TK Quinlan EM. Critical periods in amblyopia. Vis Neurosci. 2018;35:E014. 29905127 10.1017/S0952523818000020

[9] Shuai L Leilei Z Wen W. Binocular treatment in adult amblyopia is based on parvocellular or magnocellular pathway. Eur J Ophthalmol. 2020;30:658–67. 31014078 10.1177/1120672119841216

[10] Castaldi E Lunghi C Morrone MC. Neuroplasticity in adult human visual cortex. Neurosci Biobehav Rev. 2020;112:542–52. 32092315 10.1016/j.neubiorev.2020.02.028

[11] Blair K Cibis G Gulani AC. Amblyopia. StatPearls. StatPearls Publishing; 2021. 28613640

[12] Li B Zou Y Yin X. Expression of brain-derived neurotrophic factor in the lateral geniculate body of monocular form deprivation amblyopic kittens. Eur J Ophthalmol. 2021;31:2724–30. 32873060 10.1177/1120672120953341

[13] Huh CY Abdelaal K Salinas KJ. Long-term monocular deprivation during juvenile critical period disrupts binocular integration in mouse visual thalamus. J Neurosci. 2020;40:585–604. 31767678 10.1523/JNEUROSCI.1626-19.2019 PMC6961993

[14] Holmes JM Levi DM. Treatment of amblyopia as a function of age. Vis Neurosci. 2018;35:E015. 29905125 10.1017/S0952523817000220

[15] McConaghy JR McGuirk RM. Amblyopia: detection and treatment. Am Fam Physician. 2019;100:745–50. 31845774

[16] Asper L Watt K Khuu S. Optical treatment of amblyopia: a systematic review and meta-analysis. Clin Exp Optom. 2018;101:431–42. 29392811 10.1111/cxo.12657

[17] Al-Haddad C Ismail K Jurdi KW. Clinical profile and treatment outcomes of amblyopia across age groups. Middle East Afr J Ophthalmol. 2019;26:71–6. 31543663 10.4103/meajo.MEAJO_182_17 PMC6737791

[18] Papageorgiou E Asproudis I Maconachie G. The treatment of amblyopia: current practice and emerging trends. Graefes Arch Clin Exp Ophthalmol. 2019;257:1061–78. 30706134 10.1007/s00417-019-04254-w

[19] Smith EL III Hung L-F Arumugam B. Observations on the relationship between anisometropia, amblyopia and strabismus. Vision Res. 2017;134:26–42. 28404522 10.1016/j.visres.2017.03.004 PMC5499529

[20] Kraus CL Culican SM. New advances in amblyopia therapy I: binocular therapies and pharmacologic augmentation. Br J Ophthalmol. 2018;102:1492–6. 29777043 10.1136/bjophthalmol-2018-312172 PMC6241622

[21] Farvardin M Khalili MR Behnia M. Levodopa plus occlusion therapy versus occlusion therapy alone for children with anisometropic amblyopia. J Ophthalmic Vis Res. 2019;14:457–64. 31875101 10.18502/jovr.v14i4.5451 PMC6825694

[22] Tuna AR Pinto N Brardo FM. Transcranial magnetic stimulation in adults with amblyopia. J Neuroophthalmol. 2020;40:185–92. 31453915 10.1097/WNO.0000000000000828

[23] Liu Z Chen Z Xu Y. Objective assessment of the effect of optical treatment on magnocellular and parvocellular-biased visual response in anisometropic amblyopia. Invest Ophthalmol Vis Sci. 2020;61:21–21. 10.1167/iovs.61.2.21 PMC7326570 32058564

[24] Pons C Jin J Mazade R. Amblyopia affects the ON visual pathway more than the OFF. J Neurosci. 2019;39:6276–90. 31189574 10.1523/JNEUROSCI.3215-18.2019 PMC6687897

