# Supplementary material for: Analysis of the improvement in monocular amblyopia visual acuity caused by the changes in non-amblyopia visual acuity in 74 adults
Source: Medicine (Baltimore). 2023 Sep 15;102(37):e34606. doi: 10.1097/MD.0000000000034606 (PMC10508474; doi:10.1097/MD.0000000000034606)
Supplement: Supplementary file 1 [file medi-102-e34606-s001.pdf]

Supplementary Table 1. All the initial and transitioned data on amblyopia eyes, including the visual acuity, and causes of amblyopia.

Patient	Appointment time	Location of the medical appointment	Age	Initial visual acuity（best-corrected visual acuity）		Non-amblyopic eye disease	Surgery time
				Amblyopic eye	Non-amblyopic eye		
1	2018.6	Tangdu Hospital	19	0.2	0.05	Retinal detachment	2018.7
2	2018.6	Tangdu Hospital	41	0.3	Manual	Vitreous hemorrhage	2018.7
3	2018.6	Tangdu Hospital	49	0.5	0.3	Cataract	2018.9
4	2018.6	Tangdu Hospital	39	0.5	0.2	Cataract	2018.12
5	2018.7	Tangdu Hospital	53	0.5	0.1	Cataract	2019.1
6	2018.7	Tangdu Hospital	21	0.6	0.2	Cataract	2019.6
7	2018.7	Tangdu Hospital	61	0.5	0.3	Cataract	2019.5
8	2018.8	Tangdu Hospital	22	0.2	Manual	Retinal detachment	2018.8
9	2018.8	Tangdu Hospital	48	0.5	0.3	Cataract	2019.6
10	2018.9	Tangdu Hospital	43	0.6	0.2	Cataract	2019.3
11	2018.9	Tangdu Hospital	23	0.1	Index	Eye trauma	2018.10
12	2018.9	Tangdu Hospital	58	0.5	0.3	Cataract	2019.8
13	2018.10	Tangdu Hospital	62	0.6	0.2	Cataract	2019.12
14	2018.11	Tangdu Hospital	43	0.6	0.4	Cataract	2019.3
15	2018.11	Tangdu Hospital	25	0.2	0.05	Macular degeneration	2019.3
16	2018.12	Tangdu Hospital	26	0.1	0.05	Vitreous hemorrha	2019.1

						ge	
17	2018.12	Tangdu Hospital	81	0.5	0.2	Macular degeneration	2019.6
18	2019.1	Tangdu Hospital	39	0.5	0.1	Cataract	2020.3
19	2019.1	Tangdu Hospital	70	0.5	0.3	Cataract	2019.3
20	2019.1	Tangdu Hospital	57	0.4	0.1	Cataract	2019.12
21	2019.2	Tangdu Hospital	51	0.6	0.3	Cataract	2020.1
22	2019.3	Tangdu Hospital	29	0.3	Index	Vitreous hemorrhage	2019.12
23	2019.3	Tangdu Hospital	75	0.5	0.4	Cataract	2020.2
24	2019.4	Tangdu Hospital	48	0.6	0.4	Cataract	2020.12
25	2019.5	Tangdu Hospital	76	0.5	0.2	Macular degeneration	2019.12
26	2019.5	Tangdu Hospital	42	0.6	0.4	Cataract	2020.10
27	2019.5	Tangdu Hospital	37	0.6	0.4	Cataract	2021.3
28	2019.6	Tangdu Hospital	72	0.6	0.4	Cataract	2021.1
29	2019.6	Tangdu Hospital	56	0.5	0.2	Cataract	2020.2
30	2019.6	Tangdu Hospital	68	0.2	0.05	Macular degeneration	2019.10
31	2019.6	Tangdu Hospital	41	0.2	0.1	Vitreous hemorrhage	2019.8
32	2019.7	Tangdu Hospital	63	0.5	0.3	Cataract	2020.10
33	2019.7	Tangdu Hospital	36	0.2	0.1	Macular degeneration	2020.5
34	2019.8	Tangdu Hospital	58	0.4	0.2	Cataract	2020.9
35	2019.8	Tangdu Hospital	47	0.5	0.1	Cataract	2021.1
36	2019.8	Tangdu Hospital	29	0.2	0.05	Retinal detachme	2019.9

						nt	
37	2019.9	Purui ophthalmic hospital	61	0.5	0.2	Cataract	2020.9
38	2019.9	Purui ophthalmic hospital	44	0.2	0.1	Cataract	2020.8
39	2019.10	Purui ophthalmic hospital	71	0.5	0.2	Cataract	2020.7
40	2019.11	Purui ophthalmic hospital	58	0.6	0.1	Cataract	2021.5
41	2019.12	Purui ophthalmic hospital	72	0.4	0.3	Cataract	2021.6
42	2019.12	Purui ophthalmic hospital	34	0.5	0.1	Macular degeneration	2020.2
43	2019.12	Purui ophthalmic hospital	53	0.2	0.2	Cataract	2021.5
44	2020.1	Purui ophthalmic hospital	62	0.5	0.05	Cataract	2021.2
45	2020.3	Purui ophthalmic hospital	34	0.5	Index	Vitreous hemorrhage	2020.5
46	2020.3	Purui ophthalmic hospital	68	0.3	0.05	Cataract	2020.3
47	2020.3	Purui ophthalmic hospital	56	0.6	0.2	Macular degeneration	2020.5
48	2020.3	Purui ophthalmic hospital	52	0.2	0.05	Cataract	2020.5
49	2020.3	Purui ophthalmic hospital	28	0.2	0.05	Vitreous hemorrhage	2020.6
50	2020.3	Purui ophthalmic hospital	67	0.5	0.2	Cataract	2021.8
51	2020.4	Purui ophthalmic hospital	45	0.5	0.2	Cataract	2021.12
52	2020.5	Purui ophthalmic	52	0.3	0.1	Cataract	2021.10

		hospital					
53	2020.7	Purui ophthalmic hospital	46	0.4	0.2	Cataract	2021.5
54	2020.7	Purui ophthalmic hospital	35	0.2	Index	Retinal detachment	2020.7
55	2020.7	Purui ophthalmic hospital	66	0.3	0.05	Macular degeneration	2021.1
56	2020.8	Purui ophthalmic hospital	63	0.4	0.05	Macular degeneration	2020.10
57	2020.9	Purui ophthalmic hospital	56	0.5	0.2	Cataract	2021.8
58	2020.9	Purui ophthalmic hospital	53	0.4	0.2	Cataract	2021.10
59	2020.9	Purui ophthalmic hospital	34	0.3	0.1	Cataract	2021.12
60	2020.11	Purui ophthalmic hospital	36	0.2	0.1	Macular degeneration	2021.1
61	2020.12	Purui ophthalmic hospital	47	0.5	0.3	Cataract	2022.2
62	2021.1	Purui ophthalmic hospital	67	0.6	0.2	Cataract	2022.2
63	2021.3	Purui ophthalmic hospital	49	0.2	0.05	Cataract	2021.10
64	2021.3	Purui ophthalmic hospital	35	0.5	Manual	Vitreous hemorrhag	2021.3
65	2021.3	Purui ophthalmic hospital	59	0.2	0.05	Cataracte	2021.10
66	2021.3	Purui ophthalmic hospital	58	0.3	0.05	Cataract	2021.12
67	2021.3	Purui ophthalmic hospital	31	0.5	0.2	Eye trauma	2021.4
68	2021.3	Purui ophthalmic	62	0.2	0.05	Cataract	2021.9

		hospital					
69	2021.4	Purui ophthalmic hospital	52	0.2	0.1	Cataract	2022.1
70	2021.5	Purui ophthalmic hospital	68	0.5	0.05	Cataract	2022.2
71	2021.5	Purui ophthalmic hospital	33	0.3	photosensitivity	Retinal detachment	2021.5
72	2021.6	Purui ophthalmic hospital	48	0.4	0.2	Cataract	2022.12
73	2021.6	Purui ophthalmic hospital	63	0.2	Index	Macular degeneration	2021.12
74	2021.6	Purui ophthalmic hospital	51	0.5	0.05	Cataract	2022.5
